# The Gut Microbiota’s Role in Neurological, Psychiatric, and Neurodevelopmental Disorders

**DOI:** 10.3390/nu16244404

**Published:** 2024-12-22

**Authors:** Ioannis Alexandros Charitos, Angelo Michele Inchingolo, Laura Ferrante, Francesco Inchingolo, Alessio Danilo Inchingolo, Francesca Castellaneta, Antonella Cotoia, Andrea Palermo, Salvatore Scacco, Gianna Dipalma

**Affiliations:** 1 Istituti Clinici Scientifici Maugeri IRCCS, Pneumology and Respiratory Rehabilitation Unit, “Institute” of Bari, 70124 Bari, Italy; ioannis.charitos@icsmaugeri.it; 2 Interdisciplinary Department of Medicine, University of Bari “Aldo Moro”, 70124 Bari, Italy; angeloinchingolo@gmail.com (A.M.I.); lauraferrante79@virgilio.it (L.F.); ad.inchingolo@libero.it (A.D.I.); giannadipalma@tiscali.it (G.D.); 3 U.O.C. Immunohematology and Transfusion Medicine—S.I.M.T. Di Venere Hospital, 70131 Bari, Italy; francesca.castellaneta@gmail.com; 4 Department of Intensive Care, University Hospital of Foggia, 71121 Foggia, Italy; antonella.cotoia@unifg.it; 5 Department of Experimental Medicine, University of Salento, 73100 Lecce, Italy; andrea.palermo@unisalento.it; 6 Department of Translational Biomedicine and Neuroscience (DiBraiN), Aldo Moro University, 70121 Bari, Italy; salavatore.scacco@uniba.it

**Keywords:** gut microbiota (GM), neuroinflammation, gut–brain axis, neurological disorders, probiotics, autism

## Abstract

Aim: This article aims to explore the role of the human gut microbiota (GM) in the pathogenesis of neurological, psychiatric, and neurodevelopmental disorders, highlighting its influence on health and disease, and investigating potential therapeutic strategies targeting GM modulation. Materials and Methods: A comprehensive analysis of the gut microbiota’s composition and its interaction with the human body, particularly, its role in neurological and psychiatric conditions, is provided. The review discusses factors influencing GM composition, including birth mode, breastfeeding, diet, medications, and geography. Additionally, it examines the GM’s functions, such as nutrient absorption, immune regulation, and pathogen defense, alongside its interactions with the nervous system through the gut–brain axis, neurotransmitters, and short-chain fatty acids (SCFAs). Results: Alterations in the GM are linked to various disorders, including Parkinson’s disease, multiple sclerosis, depression, schizophrenia, ADHD, and autism. The GM influences cognitive functions, stress responses, and mood regulation. Antibiotic use disrupts GM diversity, increasing the risk of metabolic disorders, obesity, and allergic diseases. Emerging therapies such as probiotics, prebiotics, and microbiota transplantation show promise in modulating the GM and alleviating symptoms of neurological and psychiatric conditions. Conclusions. The modulation of the GM represents a promising approach for personalized treatment strategies. Further research is needed to better understand the underlying mechanisms and to develop targeted therapies aimed at restoring GM balance for improved clinical outcomes.

## 1. Introduction

The genetic heritage and environmental interactions of all microorganisms in a defined environment that colonize the human gastrointestinal system are defined as the gut microbiome (GMe). Instead, the term gut microbiota (GM) represents the set of all individual microorganisms, from bacteria to fungi, to protozoa to viruses [1]. The GM, along with the oral microbiota, presents the widest composition of microorganisms both qualitatively and quantitatively. Some oral diseases can affect both the airway and the GM. It is important to keep oral pathogens at the lowest possible load [2,3,4,5]. Several microorganisms have long been recognized as the causative agents of disease. Gastrointestinal pathogens such as *Salmonella* spp. or *Clostridioides difficile* are entrenched in public awareness, while research into the effects of the beneficial bacteria of the GM has been overshadowed until recently [6,7,8,9]. By increasing knowledge of its role, the GM has added a new dimension to the long-standing view that the mere presence of a single pathogenic microorganism leads to a given disease. For example, *C. difficile* can be found in up to 14% of healthy, asymptomatic individuals, while it appears to be a stable member of the eubiosi GM community [10,11,12]. In healthy individuals, the abundance and pathogenicity of the bacterium may be suppressed by the majority presence of a diverse microorganism composition the GM [13,14,15]. *C. difficile* infection is almost always preceded by broad-spectrum antibiotic therapy (usually for an unrelated issue), which causes a drastic reduction in ecosystem diversity, after which *C. difficile* can proliferate uncontrollably and become infectious [16,17,18,19]. The pathogenesis of *C. difficile* is a clear example of the importance of a dynamic GM, although there are many other studies linking abnormalities in the GM’s unhealthy microorganism diversity (dysbiosis) to a wide range of diseases [20]. In many of these cases, there is insufficient evidence to distinguish between coincidence and causation with the heterogeneity of the GM community within each individual and between individuals, making comparisons difficult. While alterations in the GM have been identified in several diseases and disorders, the physiological consequences of these changes are difficult to define, making it difficult to link them more definitively to the disease process. This is particularly true in cases of systemic diseases and may be even more so when considering psychiatric diagnoses such as schizophrenia [21,22,23,24,25]. Indeed, *C. difficile* infection can have a significant impact on feelings of anxiety and depression. However, the ability of GM to interact with the host’s nervous system could suggest a hitherto unrecognized link between the GM and neurological and psychological disease and disorders, and there is some evidence from research on both *C. difficile* and ulcerative colitis [26,27,28]. Current research suggests that alteration of the GM is associated with human health and disease, such as altered fat storage, associated with metabolic syndrome (such as obesity), cancer and other [29,30,31,32]. A pathological shift in the delicate balance of bacteria normally found in the gut encourages inflammatory responses, while beneficial bacteria (probiotics) promote anti-inflammatory properties and immune modulation [33,34]. For example, the relationship between GM, both beneficial and pathological, and autism symptomatology has been investigated in recent years, while gastrointestinal symptoms are commonly reported. Given the increasing prevalence of autism spectrum disorders and gastrointestinal problems, possibly related to pathological GM, combined with the immunological importance of vital bacteria and their importance for health, it is important to investigate the possible association between autism and the GM [35,36,37].

## 2. Methodology of Searching

The literature review was conducted over the last 10 years, between 2014 and 2024, using the scientific platforms PubMed, Scopus, and Web of Science. The search was aimed at identifying relevant studies on the interaction between GM and neurological and neuropsychiatric diseases, with a focus on the GM–brain axis (GBA). The following keywords were used: gut microbiota, GM-brain axis, dysbiosis, neurodevelopmental disorders, Parkinson’s disease, and mental health. These were combined with Boolean operators (AND, OR) to refine the results.

Inclusion criteria included original articles and reviews published in English within the last 10 years with a focus on the relationship between GM and neurological or neuropsychiatric disorders. Non-peer-reviewed articles, opinion pieces, and conference abstracts were excluded.

The search initially yielded 800 articles. After a review of titles and abstracts for relevance, 574 articles were subjected to full reading. Eventually, 228 publications were included in the review for their scientific relevance and significant contribution to the topic.

Identification:

- Records identified through database searching: 800

Screening:

- Records screened (titles and abstract): 800
- Records excluded: 226 (800–574)

Eligibility:

- Full-text articles assessed for eligibility: 574
- Full-text articles excluded (off-topic): 348

Included:

- Studies included in qualitative synthesis: 228

## 3. Objectives

The article discusses several key aspects of the GBA and its implications. Firstly, it explores the bidirectional communication between the central nervous system (CNS), autonomic nervous system (ANS), enteric nervous system (ENS), and hypothalamic–pituitary–adrenal axis (HPA), highlighting the importance of this network in influencing both mental and physical well-being. Another topic addressed is the role of the gut microbiota in producing neurotransmitters such as serotonin, dopamine, catecholamines, GABA, and ATP, demonstrating how intestinal bacteria can directly affect brain chemistry. The article delves into specific bacterial species that can modulate the GBA through the production of neurotransmitters or similar molecules, emphasizing the interconnectedness between the GM and brain functions. It also examines the link between gut dysbiosis and various neurological and psychiatric disorders, including Parkinson’s disease, multiple sclerosis, depression, schizophrenia, and bipolar disorder, showing how intestinal health can influence these pathological conditions. Lastly, attention is given to developmental neurological disorders, such as autism spectrum disorder (ASD) and attention-deficit/hyperactivity disorder (ADHD). In this context, factors related to GM, including composition and intestinal permeability, are explored as having a significant role in their development. Overall, the article covers five main research areas, offering a comprehensive and detailed overview of the importance of the GM in brain functions and neurological disorders. (1) GM Composition and Influencing Factors: exploration of how factors like diet, antibiotics, birth mode, and geography affect GM diversity and balance. (2) GBA and Biochemical Interactions: analysis of the bidirectional communication between GM and the nervous system, emphasizing the role of neurotransmitters, SCFAs, and the HPA axis in health and disease. (3) Neurological and Psychiatric Disorders: examination of GM’s role in the pathogenesis of conditions such as Parkinson’s disease, autism, ADHD, depression, and schizophrenia. (4) Therapeutic Strategies: evaluation of emerging treatments targeting GM modulation, including probiotics, prebiotics, and fecal microbiota transplantation. (5) Health Implications of GM Dysbiosis: discussion of the consequences of GM imbalances, including its links to metabolic, immune, and inflammatory disorders.

## 4. Discussion

### 4.1. Composition of Human Gut Microbiota

**Figure 1:**
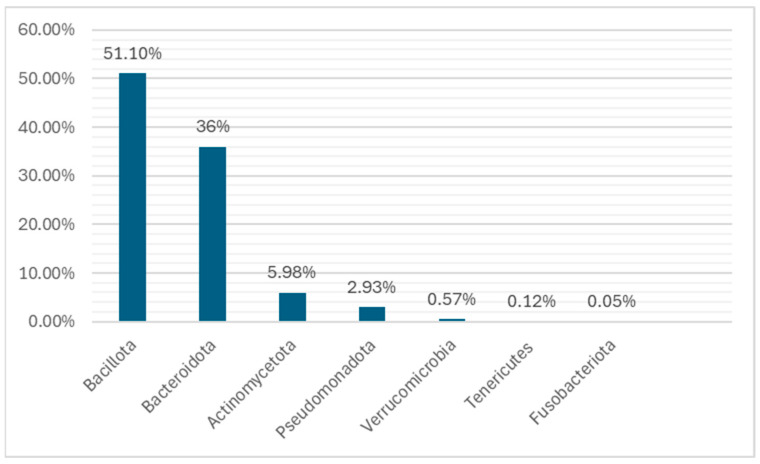
The main taxa found during childhood. These taxa and some of their species are implicated in neurological and psychiatric diseases due to their increased population in the microbiota, such as *Actinomycetota* (*Bifidobacterium* spp.), *Verrucomicrobia* (*Akkermansia* spp.), *Bacillota* (*Faecalibacterium* spp.), *Bacteroidota* (such as *Prevotella* spp.), and *Fusobacteriota* (such as *Fusobacterium* spp.). Credits: Original figure by I.A. Charitos.

**Figure 2:**
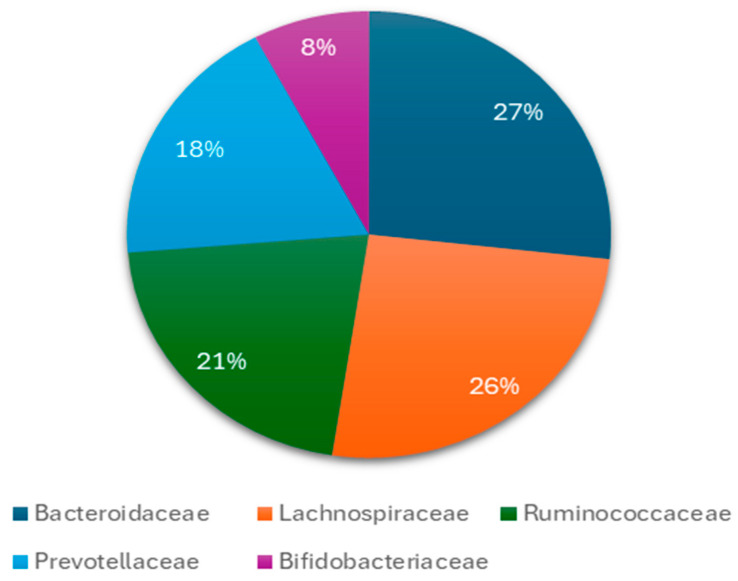
The main bacteria at level of families found during childhood in the gut microbiota. Several species from these families have a connection with neurological and psychiatric diseases or disorders such as *Bacteroides* spp., *Doria* spp., *Bifidobacteria* spp., *Prevotella* spp. and others.

The human GM is estimated to consist of 10–100 trillion microorganisms together with the genes of their cells. The totality of this genome is estimated at 3.3 million genes, compared to approximately 22,000 genes in the human body [3,38,39]. There is about 99.9% identity of the human genome, but there may be 70–90% variation among the microorganisms’ genomes of different individuals [15,40,41,42,43]. The genetic complement of the GM, or in other words, the set of “microorganisms (eukaryotic archaea, bacteria and viruses), the genetic information they carry and the environment in which they interact”, is believed to exceed that of the human genome by 100 times [4,44,45]. Until recently, the main method of isolating and recording the microorganisms of the GM was their culture [46,47,48,49,50]. the various bacteria are classified into Gram positive and Gram negative, depending on the ability of their cell wall to retain the pigment or not after treatment with alcohol [51,52,53]. The GM remains relatively stable throughout life, with average changes. Its basic constitution is formed in the early years [54,55]. The commonly accepted theory so far is that the gastrointestinal system of the newborn is sterile, but also, several studies found that the microorganisms pass to the fetus through the placenta, umbilical cord, and amniotic fluid, but this remains under debate [56,57,58]. In the first months of life, anaerobic microorganisms predominate, mainly, *Bacteroides, Bifidobacterium*, and species from the *Lactobacillaceae* family, which are classified in the phyla *Bacteroidota*, *Bacillota*, and *Actinomycetota*, respectively. *Pseudomonadota* is also added from breast milk. Studies show that approximately 28% of bacteria in infants comes from breast milk. Later, with the introduction of other different aliments (from ages 5 months to 2 years), bacteria of the *Ruminococcaceae* and *Lachnospiraceae* families, which belong to the *Bacillota* phyla, also appear in the GM. Around the age of 2 or 3 years, strains of the *Verrucomicrobia* phylum are also found. From around the age of 5, the GM of children begins to resemble that of adults (Figure 1 and Figure 2) [59,60,61,62].

**Figure 3:**
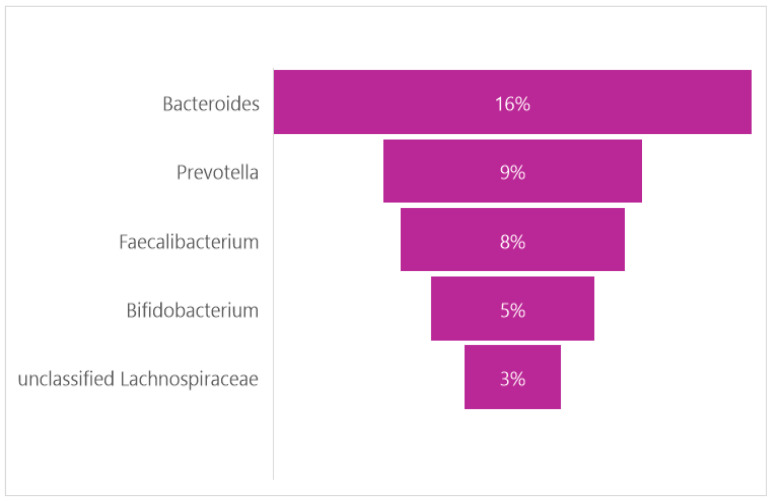
The main genera found in pediatric population.

Studies have shown that the GM of adolescents still has some differences compared to that of adults. These differences are found at the level of the bacterial genus and mainly concern *Bifidobacterium*, which appears at almost twice the percentage compared to adults (Figure 3). Credits: Original figure by I.A. Charitos [63,64,65].

**Figure 4:**
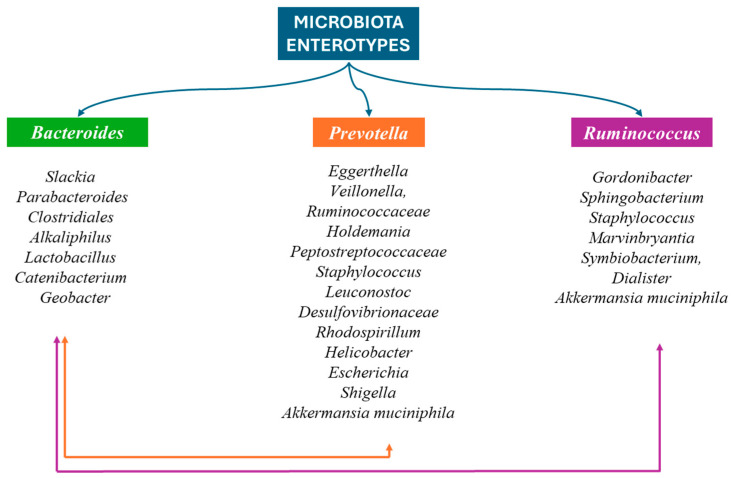
The three enterotypes are recognized based on the predominant bacterium: (1) *Bacteroides*, (2) *Prevotella*, and (3) *Ruminococcus*. In the first intestinal type, *Slackia*, *Parabacteroides*, *Clostridiales*, *Alkaliphilus*, *Lactobacillus*, *Catenibacterium*, and *Geobacter coexist*. *Eggerthella*, *Veillonella*, *Ruminococcaceae*, *Holdemania*, *Peptostreptococcaceae*, *Staphylococcus*, *Leuconostoc*, *Desulfovibrionaceae*, *Rhodospirillum*, *Helicobacter*, *Escherichia*, *Shigella*, and *Akkermansia muciniphila* also occur in the second intestinal type. Credits: Original figure by I.A. Charitos The third enteric type also includes *Gordonibacter*, *Sphingobacterium*, *Staphylococcus*, *Marvinbryantia*, *Symbiobacterium*, *Dialister*, and *Akkermansia muciniphila*. Credits: Original figure by I.A. Charitos.

In adults, the main phyla of the GM are *Bacillota* and *Bacteroidota*, which account for 90% of the GM. *Actinomycetota*, *Pseudomonadota*, *Fusobacteriota*, and *Verrucomicrobiota* are found in smaller percentages. The genus *Clostridium* accounts for 95% of the *Bacillota* and the remainder is represented by the genera *Bacillus*, *Enterococcus*, and *Ruminococcus* and the *Lactobacillaceae* family. *Bacteroidetes* consists mainly of *Bacteroides* and *Prevotella*, while the main genus of *Actinomycetota* is the *Bifidobacterium*. Everyone’s GM displays different clusters of microorganisms that shape everyone’s intestinal microbiota type. The enterotype is specific and stable for each person throughout his life, and in the case of change, it returns to the original composition. The most important difference between these types lies in the way energy is produced from the available elements of the intestinal content. The intestine types seem to be determined by eating habits, mainly during childhood age (Figure 4) [66,67,68,69].

### 4.2. The Main Factors That Affect the Gut Microbiota’s Composition

Several factors influence the composition of the GM. In neonates and infants, gestational age at birth plays a significant role. Premature birth results in delayed intestinal colonization, with limited microbiome diversity, reduced *Bifidobacterium* and *Bacteroides*, and an increase in potentially pathogenic bacteria such as *Enterobacteriaceae*. Breastfeeding is also affected by prematurity, impacting the composition of the GM [70,71,72]. The mode of birth further influences GM. During natural labor, the newborn is colonized by maternal microbiota including *Prevotella*, *Sneathia*, *Bifidobacterium longum*, and others, alongside organisms such as *Escherichia coli*, *Staphylococcus*, *Bacteroides fragilis*, and *Streptococcus*. In contrast, cesarean-section births lead to colonization by hospital-associated microorganisms like *Staphylococcus* and *Corynebacterium*, with reduced diversity and the absence of *Escherichia*, *Shigella*, and *Bacteroides* [73,74,75,76]. Diet and feeding mode also significantly affect the GM composition. Breastfeeding promotes healthier GM, characterized by increased *Bifidobacterium* spp. and decreased *C. difficile* and *E. coli*. The quality of breast milk is influenced by maternal gut health during pregnancy [77,78]. Age-related changes in diet lead to variations in GM. Adults over 70, for example, experience changes linked to dietary monotony and inflammatory conditions. Furthermore, geographic and cultural differences in diet contribute to GM variations, with diet being a key regulator of microbiota composition. Specific bacteria, including *Latilactobacillus sakei* and *Faecalibacterium prausnitzii*, are affected by diet, and obesity and BMI also play a crucial role in altering the GM composition, particularly in children. Obese children show increased *Bacillota* and decreased *Bacteroidota*, while variations in BMI result in microbiota differences [79,80,81,82,83,84]. In cases of conditions such as anorexia nervosa, significant changes in GM occur, with an increase in *Enterobacteriaceae* and *Methanobrevibacter smithii*, and a decrease in *Roseburia, Ruminococcus*, and *Clostridium* [85,86,87]. Furthermore, the use of antibiotics and xenobiotics, and drug abuse, has a substantial impact on GM. The type of antibiotic, dosage, and administration period affect microbiota composition. Macrolides, for example, decrease *Actinomycetota* and increase *Bacteroides* and *Bacillota*, while Penicillin and Clarithromycin have similar, though less severe, effects. The misuse of antibiotics, particularly in early childhood, has been linked to reduced microbiota diversity and the development of allergic diseases such as asthma and food allergies, largely influenced by specific strains of the *Clostridia genus* [88,89,90,91,92,93].

Furthermore, it has been proven that negative changes (dysbiosis) in the composition of the GM (are related also to several pathological conditions [94,95,96,97]. As we mentioned, deviations from a healthy lifestyle directly affect the GM and are related to many metabolic diseases (unhealthy gut metabolome), which, in turn, can lead to an exacerbation of symptoms or new-onset neurocognitive disorders. It must be mentioned that the first association of various diseases with the intestine is attributed to the father of medicine Hippocrates [94,98,99,100,101].

### 4.3. The GBA Biochemical Interactions

**Figure 5:**
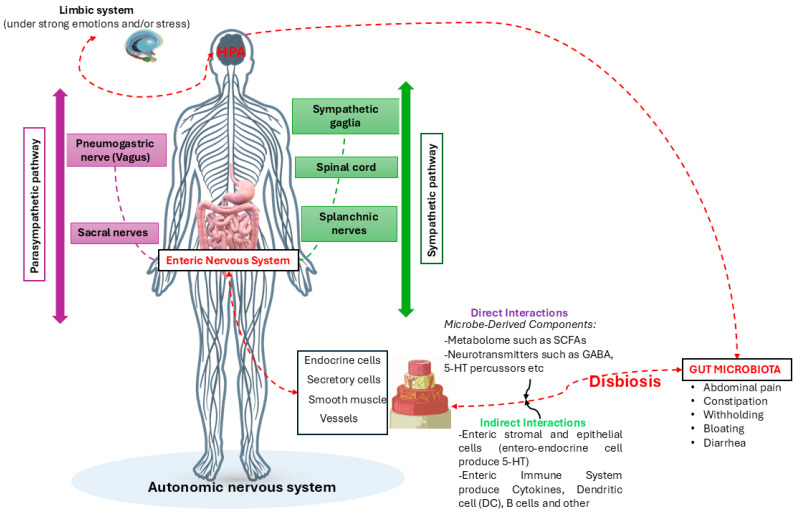
The figure describes the hypotheses of how gut dysbiosis, due to emotional or stressful causes or not, can influence the bidirectional communication of the GBA, causing direct and indirect effects on the ENS and vice versa. Credits: Original figure by I.A. Charitos.

Until recently, studies on the GBA focused on digestion and satiety. In recent years, however, emphasis has been placed on the degree of influence of higher functions, psychological state, and behavior by GBA communication [102,103,104,105]. Several research efforts are constantly being made in the direction of correlating various brain functions with GM. Animal studies more directly indicate the GBA communication [106,107,108,109,110]. The GM–brain axis includes the central nervous system (CNS), the autonomic nervous system (ANS), the enteric nervous system (ENS), the hypothalamic–pituitary–adrenal axis (HPA), and their neurotransmitters. The ANS collects information from the ENS and, through its sympathetic and parasympathetic branches, conveys it to the CNS. Conversely, it carries impulses from the CNS to the periphery. The HPA axis functions as a key regulator of the body’s response to various stressful situations [111]. The ENS is connected to the ANS, but also to the peripheral nervous system (PNS), and controls the behavior of the gastrointestinal tract, specifically, its motility, local blood flow, and absorbency, but also the secretions of the intestinal mucosa, and regulates endocrine and immune function [112,113]. Enteric neurons are connected to the ANS but do not have a direct neuronal connection with the CNS. They are organized in microcircuits, which receive stimuli from local factors and can respond to them directly, without the mediation of the CNS. Nevertheless, there is usually communication between the two systems—through neural circuits, which initially connect the ENS to the ANS and, indirectly, to the CNS—and one affects the other [114,115,116]. The ANS consists of sympathetic and parasympathetic nervous systems. The sympathetic nervous system is related to the control of gut motility, intestinal mucosal secretions, and vascular regulation by depressing the ENS and through the direct vasoconstriction of his action. The main neurotransmitters of the sympathetic nervous system are, in addition to noradrenaline (NE), neuropeptide Y (NPY) and ATP. The parasympathetic nervous system is mainly represented by the pneumogastric (or vagus) nerve [117,118,119,120]. The pneumogastric nerve is the 10th cranial nerve and its action on the gastrointestinal tract is related to food intake, digestion, the maintenance of the gastrointestinal barrier, and immunity. More specifically, the endings of the pneumogastric nerve in the intestinal mucosa act as chemoreceptors and are activated by hormones, peptides, and other elements, which release the endothelial and neuroendocrine cells of the intestine [121,122,123]. At the same time, the mechanoreceptors of the pneumogastric nerve sense the distension of the gastrointestinal tract. The result of these stimuli is causing an increase in the secretion of gastric fluids, insulin, and glucagon, with an effect on the digestion process. The pneumogastric nerve contributes to the maintenance of the intestinal barrier mainly by activating the mucus cells in the intestinal wall and strengthening the connections between them [124,125]. Finally, inflammatory mediators such as cytokines and chemical mediators from mast cells, but also several pathogens, activate the pneumogastric nerve. The result is the activation of the cholinergic anti-inflammatory pathway, in which stimuli carried by the pneumogastric nerve, mediated by acetylcholine, regulate the action of macrophages around inflammation [126,127,128]. The HPA axis is part of the limbic system, which is associated with emotion and memory. The HPA axis, with its complexity of endocrine pathways, plays a regulatory role in the response to stressful situations, but also in metabolism, immune responses, and the ANS [129,130]. In response to stressful situations, the hypothalamus secretes adrenocorticotrophic hormone-releasing factor (CRF), which causes the pituitary to release adrenocorticotropin-releasing hormone (ACTH), which leads to the secretion of cortisol from the adrenal glands [131]. Glucocorticoids that enter the circulation mobilize energy reserves, activate lipolysis and, through the mediation of the ANS, lead to vasoconstriction, and are, therefore, a consequence of the response to the stress-inducing stimulus and, therefore, to the psychological state (Figure 5) [129,132].

### 4.4. The Bacteria Neurotransmitters

An important role in the GBA connection is also played by a multitude of chemicals, which act as neurotransmitters. Many of these neurotransmitters are, in turn, linked to the GMe. Among the most important neurotransmitters are catecholamines (adrenaline, noradrenaline, and dopamine), serotonin, γ-aminobutyric acid (GABA) and adenosine triphosphatase (ATP), and histamine [133,134]. Serotonin is synthesized in the midbrain, specifically, in its nucleus seam. Recent research, however, has shown that some of it is also synthesized in intestinal nerves along the intestinal tract. Serotonin’s precursor is tryptophan, an amino acid derived from proteins that are consumed in foods such as meat, dairy, and fruit. Serotonin then binds to its various receptors with the corresponding effect [135]. Certain GM bacterial species, such as *E. coli*, are thought to be able to use tryptophan as well, indirectly reducing serotonin levels [136,137]. Besides serotonin, tryptophan is also a precursor of kynurenic acid. It seems that GM also plays a role in determining the direction that the metabolism of tryptophan will take, thus influencing the adequacy or lack of serotonin in the body [138]. Serotonin’s relationship with the gut is also indirectly demonstrated by the fact that drugs such as selective serotonin reuptake inhibitors, in addition to their action on the CNS, also affect several symptoms of chronic diseases of the gastrointestinal tract [139,140]. The basic precursor of all catecholamines is the amino acid L-tyrosine, which is first converted into L-dopa by the enzyme tyrosine hydroxylase and then into dopamine. The main regulator of the action of tyrosine hydroxylase is endogenous neuropeptide Y (NPY). Dopamine is the first catecholamine in the production chain and is mainly synthesized in the CNS [141,142]. Adrenaline and noradrenaline are synthesized either locally in the postganglionic nerve fibers of the sympathetic system or centrally in the adrenal glands. Each of the catecholamines binds to specific receptors. Noradrenaline decreases blood flow to the gut and adrenaline increases it. The action of dopamine depends on its concentration, as in low doses, it causes vasodilation, and in high doses, it causes vasoconstriction [143,144]. The participation of catecholamines in the regulation of gut motility is expressed by its reduction by adrenaline and, conversely, its increase by noradrenaline. In addition to the perfusion and motility of the intestine, adrenaline and noradrenaline also affect the absorption of nutrients [145,146]. Dopamine, on the other hand, seems to be less involved in the absorption of various substances, but is related to the reward and satisfaction system after digestion. Studies show that dopamine’s involvement in the reward system is directly regulated by stimuli from the gastrointestinal tract. New evidence shows that catecholamines are also involved in the immune mechanism of the gut, but not in the expected way. It seems that these substances reduce the immune response of the intestinal mucosa and increase the “aggressiveness” of different bacteria [147]. Adenosine triphosphatase (ATP), in addition to being a basic source of energy, also has a role as a neurotransmitter. Although its relationship to the GBA is not clear, its receptors have been identified along the intestinal tract, and in areas of gut inflammation, the concentration of ATP is increased. GABA is the main inhibitory neurotransmitter of the CNS. In the gastrointestinal tract, however, the action of GABA is reversed as it mainly causes stimulation. Its action is mainly related to intestinal peristalsis and immunity [148,149].

### 4.5. Bacteria That Affect GM–Brain Axis

Several studies conclude that GM bacteria, in addition to influencing neurotransmitters and their precursors, also secrete factors with a similar effect. For example, members of the genera *Candida*, *Streptococcus*, *Escherichia*, and *Enterococcus* have been shown to secrete serotonin, members of *Escherichia, Bacillus*, and *Saccharomyces* produce dopamine and noradrenaline, members of the *genus Lactobacillus* produce acetylcholine, and *Bifidobacterium* synthesizes GABA [150,151]. In addition to the above, the bacteria of the GM also secrete several other substances. Some of these substances, although not neurotransmitters in the strict sense of the term, act in a similar way to the nervous system. Among these substances are those from the bacteria metabolism such as short-chain fatty acids (SCFAs), with butyric, propionic, and acetic acid as main representatives, which have a particularly important position [152,153]. The SCFAs have a protective local effect on the intestine, as they contribute to its integrity, mucus production, and intestinal immunity. The blood–brain barrier is crossed by SCFAs; moreover, they are isolated in the cerebrospinal fluid, exerting a neuroprotective effect, and are possibly involved in neurodevelopmental and neurodegenerative processes [152,154].

For example, propionic acid is produced mainly by *Bacteroidota*, *Bacillota*, and *Lachnospiraceae*, while butyric acid is produced by *Faecalibacterium prausnitzii*, *Eubacterium rectale*, *Eubacterium hallii*, *Coprococcus comes*, and *Coprococcus eutactus*. In conclusion, the function of the GM–brain axis appears to be based on four mechanisms. The first is the impulses carried from the periphery to the brain via the pneumogastric nerve and spinal nerves. The following is the stimuli that are conveyed through cytokines and the various 23 types of hormones. The last mechanism is the action of the derivatives of the GM microorganisms themselves, either directly in the brain through the blood circulation, or indirectly by affecting intermediate mechanisms [155,156,157].

### 4.6. Gut Microbiota and Neurological and Psychiatric Diseases and Disorders

In recent years, the studies that associate disturbances in the intestinal microbiome and, by extension, in the gut–brain axis, with various pathological conditions are increasing. Relatively recently, interest has also shifted towards neurological, psychiatric, and neurodevelopmental disorders, which are possibly influenced by variations in the GM [158]. Suspicion of the influence of the GBA on psychiatric diseases has been around for quite some time. The relationship between stress and the GMe is bidirectional [159]. Studies have shown that stress can alter GM through the activation of the HPA and the autonomic nervous system (ANS). Conversely, it is now proven that changes in GM can cause or limit stress in the body [160,161]. Most relevant studies have been carried out in animal models. For example, it has been shown that in germ-free rodents, the addition of a specific species of *Bifidobacteria* led to increased activation of the stress-relevant stress axis. The introduction of *Lactiplantibacillus plantarum* had the opposite effect by reducing anxiety [162,163]. Parkinson’s disease is another neurological condition that is beginning to be associated with the GM composition. It is a neurodegenerative disease mainly characterized by the loss of dopaminergic neurons in the substantia nigra of the brain, accompanied by the accumulation of α-synuclein in the neurons [164,165,166]. There is now evidence to support the appearance of α-synucleinopathy (αS-pathy) already in the initial stages of the disease in the ENS [167]. Patients experience gastrointestinal disturbances due to increased intestinal permeability due to the reduced concentration of SCFAs. The composition of the GM in patients with Parkinson’s disease shows significant differences. Specifically, there is an increase mainly in *Bifidobacterium*, *Akkermansia*, *Oscillibacter*, *Alistipes*, and the Lactobacillaceae family and a decrease in *Roseburia*, *Fusicatenibacter*, and *Faecalibacterium*, which produce butyric acid and have an anti-inflammatory effect. The association of Parkinson’s disease with changes in the GM paves the way for studies in other alpha-synucleinopathies such as Alzheimer’s disease [168,169,170]. Multiple sclerosis is an inflammatory disease of the CNS of immune etiology. Of the neurological diseases that have been associated to some extent with GM, some indicative ones are mentioned [171]. Comparisons of GM in multiple sclerosis patients and healthy individuals show differences not so much in qualitative as in quantitative composition, with some populations of microorganisms appearing in greater or lesser numbers. More specifically, the population of *Akkermansia muciniphila* appears proportionally increased in patients with multiple sclerosis in recent research. The *Akkermansia* has been linked to inflammatory pathways and the activation of the complement system [172]. This connection of multiple sclerosis with the composition of the GM has opened new therapeutic avenues and related research is constantly being carried out [173]. The link between depression and bowel disorders has been identified for quite some time, although the exact mechanism has not yet been confirmed. Among the pathophysiological mechanisms that have been proposed is the one related to permeability of the intestinal mucosa. According to this mechanism, stressful situations affect the intestinal epithelial barrier, resulting in an increase in its permeability [174].

This allows Gram-negative bacteria to meet the ENS and immune cells. An immune response is induced with accumulation of inflammation factors. Accumulated cytokines lead to the stimulation of the pneumogastric nerve, which, in turn, activates the HPA axis, resulting in mood disorders [175,176,177,178]. At the same time, in patients with depression, a decrease in the diversity and differentiation of the GM and a predominance of *Bacillota*, *Bacteroidota*, and *Actinomycetota* [179,180,181] has been observed. Schizophrenia and the relationship with GM disturbances is a relatively new field of research. Inflammatory processes have been shown to be involved in the pathophysiology of the disease [182,183,184]. Disturbances in GM may alter the permeability of the intestinal wall and, thus, contribute to inflammation, through the action of cytokines [98,185]. At the same time, bacterial species that produce butyric acid have been identified in reduced concentrations in schizophrenia. Finally, the confirmed action of GM derivatives on the hematological barrier can explain the enhancement of CNS inflammation in disorders of its composition [186]. The existence of an inflammatory process of the CNS in schizophrenia has now been proven. Specific changes in the GM, such as a reduction in α-diversity markers, have been documented in patients with schizophrenia. Bacteria such as *Veillonellaceae* and *Lachnospiraceae* have been associated with disease severity. Differences in GM composition have also been identified in schizophrenia patients before and after receiving treatment [187]. Bipolar disorder affects approximately 40 million people worldwide and is mainly expressed by mood swings [188,189]. The relationship of the disease with gastrointestinal disorders such as irritable bowels has long been proven. Patients with bipolar disorder show evidence of peripheral inflammation with an increase in cytokines during disease flares. Also, inflammatory processes seem to coexist with changes in tryptophan metabolism in these patients. These changes, as already mentioned, are also related to the action of the GM [190]. More specifically, there is a decrease in the populations of microorganisms such as *Faecalibacterium*, and an increase in the populations of the *Lactobacillaceae* family and *Enterococcus*, as well as *Actinomycetota phyla*. At the same time, the pharmaceutical preparations used to treat bipolar disorder have also been shown to affect the balance between the individual populations of GM. In this way, the relationship between the change in the composition of the GM and the manifestation of bipolar disorder is indirectly demonstrated [191].

### 4.7. Gut Microbiota and Neurodevelopmental Disorders

The neurodevelopmental disorders include ASD, intellectual disability, attention deficit hyperactivity disorder (ADHD), communication disorders, developmental motor disorders including tics, and specific learning disabilities [192]. The most sensitive periods for the appearance of neurodevelopmental disorders are considered the fetal period, childbirth, the breastfeeding period, and, in general infancy. During this period, factors such as stress, eating habits, and the mother’s endocrinological and immune status, but also the composition of the mother’s gut and vaginal microbiota, as well as the route of delivery, can affect the development of the child’s CNS, leading or not to the appearance of neurodevelopmental disorders [193,194]. In ADHD, hypermobility with changes in the composition of the GM appeared in the literature much more recently, and mainly after the corresponding studies concerning autism spectrum disorders. Most of these theories are since several conditions associated with the onset of ADHD in a person are also associated with changes in the GM. Given the function of the GBA, as already mentioned, a theoretical etiological model for ADHD is created [195,196,197]. More specifically, the relationship between the mode of birth and ADHD has already been suggested through the increased rates of its occurrence in people born by the caesarean section. Research indicates that children born by caesarean section may have a higher risk of developing ADHD compared to those born via vaginal delivery. The mode of birth affects the initial colonization of the newborn’s gut microbiota, which plays a crucial role in neurodevelopment [198,199,200]. Infants are exposed to different microbial environments than those born vaginally, potentially impacting the gut–brain axis. Additionally, the stress response during vaginal delivery triggers the release of hormones that aid brain development, a process that might be less pronounced in caesarean section births. Perinatal factors, such as complications during labor that necessitate emergency CS, are also linked to an increased risk of ADHD. While there is evidence suggesting a link between caesarean section and ADHD, the relationship is complex and influenced by multiple factors, necessitating further research to clarify the underlying mechanisms. Increased rates of the disorder are also found in people who were born prematurely, as well as in people who did not breastfeed in their newborn age. There are also studies linking ADHD to taking certain antibiotics at a young age [200,201,202,203]. However, these studies are not proof of the relationship, as there is a large degree of discrepancy between them. Indeed, in a cohort study in twins suggests that there is no association between ADHD and ASD diagnoses and early-life antibiotic use when environmental and genetic family factors are considered. Finally, in a population-based cohort study, the results suggest that early life antibiotic exposure has minimal impact on the risk of ASD and/or ADHD. Thus, the risk increase should not postpone reasonable antibiotic use. What could be taken as a conclusion is that the age of taking antibiotics plays a role in the later onset of ADHD, with a greater burden at younger ages. All the conditions are also related to changes in GM, thus raising the question of the association of ADHD with these changes [204,205,206] (Table 1).

Of all the neurodevelopmental disorders, the appearance of ASD has been most extensively linked to the presence of changes in GM. Years of observations have linked gastrointestinal disorders to autism. More specifically, increased incidence rates of diarrheal stools, constipation, flatulence, etc. have been demonstrated. The link between autism and inflammatory bowel disease has also been described in these individuals, which is called “autistic enterocolitis” [207]. At the same time, disturbances in the permeability of the intestine have been observed in people with ASD, resulting in the increased absorption of pathogenic substances such as endotoxemia with neurotoxins. Changes in the composition and balance among the various microorganisms of the GM appear to be related to the ASD phenotype [208,209]. A series of studies and meta-analyses in animal models and in humans in recent years has tried to prove the existence of this relationship, with particularly encouraging results. Regarding the composition of the GM, studies have shown an increase in the populations of *Bacillota* phyla, compared to those of *Bacteroidota phyla*, with a simultaneous decrease in *Alistipes*, *Bilophila*, *Prevotella*, and *Veillonella* and a significant increase in *Collinsella*, *Corynebacterium*, and the lactobacillaceae family in individuals with ASD. An increase has also been observed in the microbial populations of *Clostridium* and *Akkermansia* species in children with ASD compared to normal individuals [210,211,212]. Also, according to one study, bacteria of the genus *Desulfovibrio* were detected in almost half of the subjects with ASD and almost none in the normal subjects. Although the existence and, even more so, the nature, of a causal relationship between changes in the GM and autism has not been proven with certainty, several theories have been proposed in this direction [213]. Most theories concerning environmental influences focus on the fetal and perinatal period. Infections during pregnancy, and mainly, febrile infections with antipyretics, have been described in the prenatal history of individuals with autism [214,215]. Also, caesarean birth has been confirmed in a percentage of around 12–13% of people with ASD. This mode of birth alters the microbial colonization of the newborn, as instead of the normal microorganism of the mother’s vagina, the newborn is colonized mainly with skin microbiota [216]. The mode of birth affects the newborn’s initial microbiota colonization. Babies born by caesarean section are primarily colonized with skin microbiota, which can lead to GM imbalances (dysbiosis). This dysbiosis may influence neurodevelopment and is associated with an increased risk of ASD. The altered microbiota can affect gut permeability, immune system development, and the production of microbial metabolites, potentially contributing to ASD symptoms. Further research is needed to understand these mechanisms fully. Reference is also made to the administration of antibiotic substances both to the mother during pregnancy and to the newborn in the perinatal period. Antibiotic use has been shown to alter the GM, but there is insufficient evidence to demonstrate the permanence of these changes [217]. Alterations in the GM in individuals with ASD may cause rivalries between different strains, resulting in a reduction in microorganism diversity and function in the GM. Various metabolites and/or conjugates/derivatives of the microorganisms may change their action with the change in diversity in the GM, or the GM may be weakened, resulting in the appearance of the corresponding symptoms [218,219]. For example, the *Clostridia* species produces a substance, namely, 3-(3-hydroxyphenyl)-3 hydroxypropionic acid, which is considered harmful, as it reduces the concentrations of catecholamines. This substance is found to be elevated in autism and is blamed for the appearance of symptoms such as stereotypies in animal research models. Studies linking autism to the GM have paved the way for therapeutic interventions aimed at the GM could possibly reduce autism symptoms [220]. Probiotics are live bacteria that are found in various foods and their intake has several positive effects on the body. Specific species of probiotics, such as *Bacteroides fragilis*, have been shown in animal studies to improve behavioral disturbances, stereotypies, and anxiety symptoms often seen in ASD. Similar results are obtained from studies also in animals with the administration of some species of the *Lactobacillaceae* family [221,222,223]. Prebiotics are non-digestible nutritional substances, which serve as “food” for the non-pathogenic strains of the GM. Administration of prebiotics to people with autism, usually in combination with probiotics, had positive effects on the symptoms of the disorder. Taking certain antibiotics, such as vancomycin and metronidazole, had similar results, probably due to the limitation of PathoBact in the intestinal tract [224,225]. A more ambitious approach to treating ASD through the GM is transfer therapy and the transplantation of fecal microorganism (fecal microbial transplant) [226,227,228]. Although numerous studies have suggested an association between alterations in the GM and the symptoms of ASD, there is still no conclusive evidence demonstrating a direct causal link in humans, so further research is required.

## 5. Conclusions

The present study highlights several key findings regarding the GBA, which refers to the interactions between the central nervous system, the gastrointestinal system, and the microorganisms that live in the intestinal tract. Existing studies, including those in pediatric populations such as children with autism, suggest that changes in the GM could indeed be related to gastrointestinal disorders. This provides a rationale for why gastrointestinal disorders commonly occur in children with autism. Specific bacterial taxa, such as increased populations of *Bacillota* and decreased populations of *Bacteroidota*, have been identified in individuals with ASD. Other notable changes include increased levels of *Clostridium* and *Akkermansia* species. While similar changes in GM composition have been observed in ADHD, the evidence is less consistent. Further research is needed to determine if the same bacterial species are associated with both ASD and ADHD. GM dysbiosis can lead to conditions affecting mood, causing anxiety, depression, and other neuropsychiatric symptoms. While numerous studies suggest associations between GM changes and neurodevelopmental disorders, definitive causal relationships, especially in humans, have not been established. Many findings are based on animal models, and extrapolating these results to humans requires caution. The GM shows variability from person to person under normal conditions. Large-scale systematic studies with clinical correlations are needed to understand how to effectively modify GM for therapeutic purposes. Overall, the study of the GM holds promise for future therapeutic and diagnostic possibilities, including the use of potential biomarkers in various diseases and disorders. However, further research is essential to translate these findings into clinical practice.

## Figures and Tables

**Table 1:** Different disorders, the type of GM involved, and the main results with relevant citations.

Disorder	Type of GM Involved	Main Findings
Parkinson’s disease	*Bifidobacterium*, *Akkermansia*, *Oscillibacter*, *Alistipes*, *Lactobacillaceae*, *Roseburia*, *Fusicanibacter*, *Faecalibacterium*	Increased intestinal permeability due to reduced concentration of SCFAs; significant differences in GM composition; increased *Bifidobacterium*, *Akkermansia*, *Oscillibacter*, *Alistipes*, *Lactobacillaceae*; decreased *Roseburia*, *Fusicanibacter*, *Faecalibacterium* [175,181].
Multiple sclerosis	*Akkermansia muciniphila*	Proportional increase in *Akkermansia muciniphila*; linked to inflammatory pathways and activation of the complement system [182,183,184].
Depression	*Bacillota*, *Bacteroidota*, *Actinomycetota*	Decreased diversity and differentiation of GM; predominance of *Bacillota*, *Bacteroidota*, *Actinomycetota*; increased intestinal permeability leading to immune response and mood disorders [188,189,192].
Schizophrenia	*Veillonellaceae*, *Lachnospiraceae*	Reduced concentrations of butyric acid-producing bacteria; specific changes in GM such as reduction in α-diversity markers; association with disease severity; differences in GM composition before and after treatment [195,196,197].
Bipolar disorder	*Faecalibacterium*, *Lactobacillaceae*, *Enterococcus*, *Actinomycetota*	Decrease in *Faecalibacterium*; increase in *Lactobacillaceae*, *Enterococcus*, *Actinomycetota*; pharmaceutical treatments affect GM balance [198,199,200,201].

## Data Availability

The original contributions presented in this study are included in the article. Further inquiries can be directed to the corresponding author.

## Abbreviations

ACE	Angiotensin-Converting Enzyme
ACTH	adrenocorticotropin-releasing hormone
ADHD	attention deficit hyperactivity disorder
ANS	Autonomic Nervous System
ASD	Autism spectrum disorders
ATP	adenosine triphosphatase
BMI	body mass index
CNS	Central Nervous System
CRF	adrenocorticotrophic hormone-releasing factor
DSM	Diagnostic and Statistical Manual of Mental Disorders
ENS	Enteric Nervous System
GABA	Gamma-aminobutyric acid
GBA	gut-brain axis
GMe	Gut microbiome
GM	Gut Microbiota
HPA	Hypothalamic-Pituitary-Adrenal Axis
NE	Noradrenaline
NP	neuropeptide
OS	Operational Species
OUT	Operational Taxonomic Unit
PNS	Peripheral Nervous System
RNA	Ribonucleic Acid
SCFAs	short chain fatty acids

## References

[1] Colella M., Charitos I.A., Ballini A., Cafiero C., Topi S., Palmirotta R., Santacroce L. (2023). Microbiota Revolution: How Gut Microbes Regulate Our Lives. World J. Gastroenterol..

[2] Santacroce L., Passarelli P.C., Azzolino D., Bottalico L., Charitos I.A., Cazzolla A.P., Colella M., Topi S., Godoy F.G., D’Addona A. (2023). Oral Microbiota in Human Health and Disease: A Perspective. Exp. Biol. Med. Maywood.

[3] Ursell L.K., Metcalf J.L., Parfrey L.W., Knight R. (2012). Defining the Human Microbiome. Nutr. Rev..

[4] Thursby E., Juge N. (2017). Introduction to the Human Gut Microbiota. Biochem. J..

[5] Bengmark S. (1998). Ecological Control of the Gastrointestinal Tract. The Role of Probiotic Flora. Gut.

[6] Sehgal K., Khanna S. (2021). Gut Microbiome and Clostridioides Difficile Infection: A Closer Look at the Microscopic Interface. Ther. Adv. Gastroenterol..

[7] Seekatz A.M., Safdar N., Khanna S. (2022). The Role of the Gut Microbiome in Colonization Resistance and Recurrent Clostridioides Difficile Infection. Ther. Adv. Gastroenterol..

[8] Lavoie T., Appaneal H.J., LaPlante K.L. (2023). Advancements in Novel Live Biotherapeutic Products for Clostridioides Difficile Infection Prevention. Clin. Infect. Dis..

[9] Davis C.D. (2016). The Gut Microbiome and Its Role in Obesity. Nutr. Today.

[10] Ozaki E., Kato H., Kita H., Karasawa T., Maegawa T., Koino Y., Matsumoto K., Takada T., Nomoto K., Tanaka R. (2004). Clostridium Difficile Colonization in Healthy Adults: Transient Colonization and Correlation with Enterococcal Colonization. J. Med. Microbiol..

[11] Fredheim E.G.A., Klingenberg C., Rohde H., Frankenberger S., Gaustad P., Flaegstad T., Sollid J.E. (2009). Biofilm Formation by Staphylococcus Haemolyticus. J. Clin. Microbiol..

[12] Berg R.D. (1995). Bacterial Translocation from the Gastrointestinal Tract. Trends Microbiol..

[13] Van den Abbeele P., Belzer C., Goossens M., Kleerebezem M., De Vos W.M., Thas O., De Weirdt R., Kerckhof F.-M., Van de Wiele T. (2013). Butyrate-Producing Clostridium Cluster XIVa Species Specifically Colonize Mucins in an in Vitro Gut Model. ISME J..

[14] Inchingolo F., Tatullo M., Abenavoli F.M., Marrelli M., Inchingolo A.D., Villabruna B., Inchingolo A.M., Dipalma G. (2010). Severe Anisocoria after Oral Surgery under General Anesthesia. Int. J. Med. Sci..

[15] Aggarwal N., Kitano S., Puah G.R.Y., Kittelmann S., Hwang I.Y., Chang M.W. (2023). Microbiome and Human Health: Current Understanding, Engineering, and Enabling Technologies. Chem. Rev..

[16] Dethlefsen L., Huse S., Sogin M.L., Relman D.A. (2008). The Pervasive Effects of an Antibiotic on the Human Gut Microbiota, as Revealed by Deep 16S rRNA Sequencing. PLoS Biol..

[17] Mada P.K., Alam M.U. Figure, Impression Smear of Clostridioides Difficile. https://www.ncbi.nlm.nih.gov/books/NBK431054/figure/article-19632.image.f3/.

[18] Corriero A., Giglio M., Soloperto R., Inchingolo F., Varrassi G., Puntillo F. (2024). Microbial Symphony: Exploring the Role of the Gut in Osteoarthritis-Related Pain. A Narrative Review. Pain Ther..

[19] Dethlefsen L., McFall-Ngai M., Relman D.A. (2007). An Ecological and Evolutionary Perspective on Human-Microbe Mutualism and Disease. Nature.

[20] Bien J., Palagani V., Bozko P. (2013). The Intestinal Microbiota Dysbiosis and Clostridium Difficile Infection: Is There a Relationship with Inflammatory Bowel Disease?. Ther. Adv. Gastroenterol..

[21] von Bartheld C.S. (2018). Myths and Truths about the Cellular Composition of the Human Brain: A Review of Influential Concepts. J. Chem. Neuroanat..

[22] Topi S., Bottalico L., Charitos I.A., Colella M., Di Domenico M., Palmirotta R., Santacroce L. (2022). Biomolecular Mechanisms of Autoimmune Diseases and Their Relationship with the Resident Microbiota: Friend or Foe?. Pathophysiology.

[23] Dipalma G., Inchingolo A.D., Inchingolo F., Charitos I.A., Di Cosola M., Cazzolla A.P. (2021). Focus on the Cariogenic Process: Microbial and Biochemical Interactions with Teeth and Oral Environment. J. Biol. Regul. Homeost. Agents.

[24] Imfeld T. (2008). Nutrition, diet and dental health--de- and remineralisation of teeth. Ther. Umsch. Rev. Ther..

[25] Abrams G.D. (1988). Pathogenesis of Gastrointestinal Infections. Am. J. Surg. Pathol..

[26] Ullah H., Arbab S., Tian Y., Liu C., Chen Y., Qijie L., Khan M.I.U., Hassan I.U., Li K. (2023). The Gut Microbiota–Brain Axis in Neurological Disorder. Front. Neurosci..

[27] Abdel-Haq R., Schlachetzki J.C.M., Glass C.K., Mazmanian S.K. (2019). Microbiome–Microglia Connections via the Gut–Brain Axis. J. Exp. Med..

[28] Alaish S.M., Smith A.D., Timmons J., Greenspon J., Eyvazzadeh D., Murphy E., Shea-Donahue T., Cirimotich S., Mongodin E., Zhao A. (2013). Gut Microbiota, Tight Junction Protein Expression, Intestinal Resistance, Bacterial Translocation and Mortality Following Cholestasis Depend on the Genetic Background of the Host. Gut Microbes.

[29] Bäckhed F., Manchester J.K., Semenkovich C.F., Gordon J.I. (2007). Mechanisms Underlying the Resistance to Diet-Induced Obesity in Germ-Free Mice. Proc. Natl. Acad. Sci. USA.

[30] Fahed G., Aoun L., Bou Zerdan M., Allam S., Bou Zerdan M., Bouferraa Y., Assi H.I. (2022). Metabolic Syndrome: Updates on Pathophysiology and Management in 2021. Int. J. Mol. Sci..

[31] Prasun P. (2020). Mitochondrial Dysfunction in Metabolic Syndrome. Biochim. Biophys. Acta Mol. Basis Dis..

[32] Inchingolo F., Inchingolo A.D., Palumbo I., Trilli I., Guglielmo M., Mancini A., Palermo A., Inchingolo A.M., Dipalma G. (2024). The Impact of Cesarean Section Delivery on Intestinal Microbiota: Mechanisms, Consequences, and Perspectives—A Systematic Review. Int. J. Mol. Sci..

[33] Wu H.-J., Wu E. (2012). The Role of Gut Microbiota in Immune Homeostasis and Autoimmunity. Gut Microbes.

[34] Inchingolo F., Inchingolo A.M., Avantario P., Settanni V., Fatone M.C., Piras F., Di Venere D., Inchingolo A.D., Palermo A., Dipalma G. (2023). The Effects of Periodontal Treatment on Rheumatoid Arthritis and of Anti-Rheumatic Drugs on Periodontitis: A Systematic Review. Int. J. Mol. Sci..

[35] Alharthi A., Alhazmi S., Alburae N., Bahieldin A. (2022). The Human Gut Microbiome as a Potential Factor in Autism Spectrum Disorder. Int. J. Mol. Sci..

[36] Chernikova M.A., Flores G.D., Kilroy E., Labus J.S., Mayer E.A., Aziz-Zadeh L. (2021). The Brain-Gut-Microbiome System: Pathways and Implications for Autism Spectrum Disorder. Nutrients.

[37] Saurman V., Margolis K.G., Luna R.A. (2020). Autism Spectrum Disorder as a Brain-Gut-Microbiome Axis Disorder. Dig. Dis. Sci..

[38] Kwong W.K., Moran N.A. (2015). Evolution of Host Specialization in Gut Microbes: The Bee Gut as a Model. Gut Microbes.

[39] Corriero A., Giglio M., Inchingolo F., Moschetta A., Varrassi G., Puntillo F. (2024). Gut Microbiota Modulation and Its Implications on Neuropathic Pain: A Comprehensive Literature Review. Pain Ther..

[40] Logsdon G.A., Vollger M.R., Eichler E.E. (2020). Long-Read Human Genome Sequencing and Its Applications. Nat. Rev. Genet..

[41] Pottie I., Vázquez Fernández R., Van de Wiele T., Briers Y. (2024). Phage Lysins for Intestinal Microbiome Modulation: Current Challenges and Enabling Techniques. Gut Microbes.

[42] Brüssow H. (2017). Infection Therapy: The Problem of Drug Resistance- and Possible Solutions. Microb. Biotechnol..

[43] Inchingolo F., Inchingolo A.M., Malcangi G., De Leonardis N., Sardano R., Pezzolla C., de Ruvo E., Di Venere D., Palermo A., Inchingolo A.D. (2023). The Benefits of Probiotics on Oral Health: Systematic Review of the Literature. Pharmaceuticals.

[44] Lin L., Zhang J. (2017). Role of Intestinal Microbiota and Metabolites on Gut Homeostasis and Human Diseases. BMC Immunol..

[45] Tanemoto S., Sujino T., Kanai T. (2017). Intestinal immune response is regulated by gut microbe. Nihon Rinsho Meneki Gakkai Kaishi.

[46] Wang Z., Zolnik C.P., Qiu Y., Usyk M., Wang T., Strickler H.D., Isasi C.R., Kaplan R.C., Kurland I.J., Qi Q. (2018). Comparison of Fecal Collection Methods for Microbiome and Metabolomics Studies. Front. Cell. Infect. Microbiol..

[47] Flores R., Shi J., Yu G., Ma B., Ravel J., Goedert J.J., Sinha R. (2015). Collection Media and Delayed Freezing Effects on Microbial Composition of Human Stool. Microbiome.

[48] Walters K.E., Martiny J.B.H. (2020). Alpha-, Beta-, and Gamma-Diversity of Bacteria Varies across Habitats. PLoS ONE.

[49] Fierer N., Leff J.W., Adams B.J., Nielsen U.N., Bates S.T., Lauber C.L., Owens S., Gilbert J.A., Wall D.H., Caporaso J.G. (2012). Cross-Biome Metagenomic Analyses of Soil Microbial Communities and Their Functional Attributes. Proc. Natl. Acad. Sci. USA.

[50] Inchingolo A.M., Patano A., Piras F., Mancini A., Inchingolo A.D., Paduanelli G., Inchingolo F., Palermo A., Dipalma G., Malcangi G. (2023). Interconnection between Microbiota-Gut-Brain Axis and Autism Spectrum Disorder Comparing Therapeutic Options: A Scoping Review. Microorganisms.

[51] Kim B.-R., Shin J., Guevarra R., Lee J.H., Kim D.W., Seol K.-H., Lee J.-H., Kim H.B., Isaacson R. (2017). Deciphering Diversity Indices for a Better Understanding of Microbial Communities. J. Microbiol. Biotechnol..

[52] Thomas A.M., Jesus E.C., Lopes A., Aguiar S., Begnami M.D., Rocha R.M., Carpinetti P.A., Camargo A.A., Hoffmann C., Freitas H.C. (2016). Tissue-Associated Bacterial Alterations in Rectal Carcinoma Patients Revealed by 16S rRNA Community Profiling. Front. Cell. Infect. Microbiol..

[53] Li G., Yang M., Zhou K., Zhang L., Tian L., Lv S., Jin Y., Qian W., Xiong H., Lin R. (2015). Diversity of Duodenal and Rectal Microbiota in Biopsy Tissues and Luminal Contents in Healthy Volunteers. J. Microbiol. Biotechnol..

[54] Zhang Y., Wei C., Guo C.-C., Bi R.-X., Xie J., Guan D.-H., Yang C.-H., Jiang Y.-H. (2017). Prognostic Value of microRNAs in Hepatocellular Carcinoma: A Meta-Analysis. Oncotarget.

[55] Li J., Lin C., Zhou X., Zhong F., Zeng P., Yang Y., Zhang Y., Yu B., Fan X., McCormick P.J. (2022). Structural Basis of the Main Proteases of Coronavirus Bound to Drug Candidate PF-07321332. J. Virol..

[56] Santacroce L., Man A., Charitos I.A., Haxhirexha K., Topi S. (2021). Current Knowledge about the Connection between Health Status and Gut Microbiota from Birth to Elderly. A Narrative Review. Front. Biosci. Landmark Ed..

[57] Russo R., Cristiano C., Avagliano C., De Caro C., La Rana G., Raso G.M., Canani R.B., Meli R., Calignano A. (2018). Gut-Brain Axis: Role of Lipids in the Regulation of Inflammation, Pain and CNS Diseases. Curr. Med. Chem..

[58] Sun M.-F., Shen Y.-Q. (2018). Dysbiosis of Gut Microbiota and Microbial Metabolites in Parkinson’s Disease. Ageing Res. Rev..

[59] Yao Y., Cai X., Ye Y., Wang F., Chen F., Zheng C. (2021). The Role of Microbiota in Infant Health: From Early Life to Adulthood. Front. Immunol..

[60] Goodrich J.K., Davenport E.R., Beaumont M., Jackson M.A., Knight R., Ober C., Spector T.D., Bell J.T., Clark A.G., Ley R.E. (2016). Genetic Determinants of the Gut Microbiome in UK Twins. Cell Host Microbe.

[61] Kreutz A., Chang X., Hogberg H.T., Wetmore B.A. (2024). Advancing Understanding of Human Variability through Toxicokinetic Modeling, in Vitro-in Vivo Extrapolation, and New Approach Methodologies. Hum. Genom..

[62] Hiruki T., Fernandes B., Ramsay J., Rother I. (1992). Acute Typhlitis in an Immunocompromised Host. Report of an Unusual Case and Review of the Literature. Dig. Dis. Sci..

[63] Agans R., Rigsbee L., Kenche H., Michail S., Khamis H.J., Paliy O. (2011). Distal Gut Microbiota of Adolescent Children Is Different from That of Adults. FEMS Microbiol. Ecol..

[64] Rigsbee L., Agans R., Shankar V., Kenche H., Khamis H.J., Michail S., Paliy O. (2012). Quantitative Profiling of Gut Microbiota of Children with Diarrhea-Predominant Irritable Bowel Syndrome. Am. J. Gastroenterol..

[65] Malcangi G., Patano A., Ciocia A.M., Netti A., Viapiano F., Palumbo I., Trilli I., Guglielmo M., Inchingolo A.D., Dipalma G. (2023). Benefits of Natural Antioxidants on Oral Health. Antioxidants.

[66] Arumugam M., Raes J., Pelletier E., Le Paslier D., Yamada T., Mende D.R., Fernandes G.R., Tap J., Bruls T., Batto J.-M. (2011). Enterotypes of the Human Gut Microbiome. Nature.

[67] Liang C., Tseng H.-C., Chen H.-M., Wang W.-C., Chiu C.-M., Chang J.-Y., Lu K.-Y., Weng S.-L., Chang T.-H., Chang C.-H. (2017). Diversity and Enterotype in Gut Bacterial Community of Adults in Taiwan. BMC Genom..

[68] de Moraes A.C.F., Fernandes G.R., da Silva I.T., Almeida-Pititto B., Gomes E.P., Pereira A.d.C., Ferreira S.R.G. (2017). Enterotype May Drive the Dietary-Associated Cardiometabolic Risk Factors. Front. Cell. Infect. Microbiol..

[69] Turnbaugh P.J., Ley R.E., Hamady M., Fraser-Liggett C.M., Knight R., Gordon J.I. (2007). The Human Microbiome Project. Nature.

[70] Beharry K.D., Latkowska M., Valencia A.M., Allana A., Soto J., Cai C.L., Golombek S., Hand I., Aranda J.V. (2023). Factors Influencing Neonatal Gut Microbiome and Health with a Focus on Necrotizing Enterocolitis. Microorganisms.

[71] Inchingolo A.M., Gargiulo Isacco C., Inchingolo A.D., Nguyen K.C.D., Cantore S., Santacroce L., Scacco S., Cirulli N., Corriero A., Puntillo F. (2023). The Human Microbiota Key Role in the Bone Metabolism Activity. Eur. Rev. Med. Pharmacol. Sci..

[72] Gregory K.E., Samuel B.S., Houghteling P., Shan G., Ausubel F.M., Sadreyev R.I., Walker W.A. (2016). Influence of Maternal Breast Milk Ingestion on Acquisition of the Intestinal Microbiome in Preterm Infants. Microbiome.

[73] Suárez-Martínez C., Santaella-Pascual M., Yagüe-Guirao G., Martínez-Graciá C. (2023). Infant Gut Microbiota Colonization: Influence of Prenatal and Postnatal Factors, Focusing on Diet. Front. Microbiol..

[74] Adamczak A.M., Werblińska A., Jamka M., Walkowiak J. (2024). Maternal-Foetal/Infant Interactions-Gut Microbiota and Immune Health. Biomedicines.

[75] Inchingolo A.D., Dipalma G., Palmieri G., Di Pede C., Semjonova A., Patano A., Ceci S., Cardarelli F., Montenegro V., Garibaldi M. (2022). Functional Breastfeeding: From Nutritive Sucking to Oral Health. J. Biol. Regul. Homeost. Agents.

[76] Luckey T.D. (1972). Introduction to Intestinal Microecology. Am. J. Clin. Nutr..

[77] Fouhy F., Ross R.P., Fitzgerald G.F., Stanton C., Cotter P.D. (2012). Composition of the Early Intestinal Microbiota: Knowledge, Knowledge Gaps and the Use of High-Throughput Sequencing to Address These Gaps. Gut Microbes.

[78] Kelsen J.R., Wu G.D. (2012). The Gut Microbiota, Environment and Diseases of Modern Society. Gut Microbes.

[79] Bolte L.A., Vich Vila A., Imhann F., Collij V., Gacesa R., Peters V., Wijmenga C., Kurilshikov A., Campmans-Kuijpers M.J.E., Fu J. (2021). Long-Term Dietary Patterns Are Associated with pro-Inflammatory and Anti-Inflammatory Features of the Gut Microbiome. Gut.

[80] Turpin W., Dong M., Sasson G., Raygoza Garay J.A., Espin-Garcia O., Lee S.-H., Neustaeter A., Smith M.I., Leibovitzh H., Guttman D.S. (2022). Mediterranean-Like Dietary Pattern Associations With Gut Microbiome Composition and Subclinical Gastrointestinal Inflammation. Gastroenterology.

[81] Zhang Z., Taylor L., Shommu N., Ghosh S., Reimer R., Panaccione R., Kaur S., Hyun J.E., Cai C., Deehan E.C. (2020). A Diversified Dietary Pattern Is Associated With a Balanced Gut Microbial Composition of Faecalibacterium and Escherichia/Shigella in Patients With Crohn’s Disease in Remission. J. Crohns Colitis.

[82] Houtman T.A., Eckermann H.A., Smidt H., de Weerth C. (2022). Gut Microbiota and BMI throughout Childhood: The Role of Firmicutes, Bacteroidetes, and Short-Chain Fatty Acid Producers. Sci. Rep..

[83] Zhang S., Dang Y. (2022). Roles of Gut Microbiota and Metabolites in Overweight and Obesity of Children. Front. Endocrinol..

[84] Inchingolo A.M., Patano A., Di Pede C., Inchingolo A.D., Palmieri G., de Ruvo E., Campanelli M., Buongiorno S., Carpentiere V., Piras F. (2023). Autologous Tooth Graft: Innovative Biomaterial for Bone Regeneration. Tooth Transformer^®^ and the Role of Microbiota in Regenerative Dentistry. A Systematic Review. J. Funct. Biomater..

[85] Borgo F., Riva A., Benetti A., Casiraghi M.C., Bertelli S., Garbossa S., Anselmetti S., Scarone S., Pontiroli A.E., Morace G. (2017). Microbiota in Anorexia Nervosa: The Triangle between Bacterial Species, Metabolites and Psychological Tests. PLoS ONE.

[86] Prochazkova P., Roubalova R., Dvorak J., Kreisinger J., Hill M., Tlaskalova-Hogenova H., Tomasova P., Pelantova H., Cermakova M., Kuzma M. (2021). The Intestinal Microbiota and Metabolites in Patients with Anorexia Nervosa. Gut Microbes.

[87] Yu D., Nguyen S.M., Yang Y., Xu W., Cai H., Wu J., Cai Q., Long J., Zheng W., Shu X.-O. (2021). Long-Term Diet Quality Is Associated with Gut Microbiome Diversity and Composition among Urban Chinese Adults. Am. J. Clin. Nutr..

[88] Kesavelu D., Jog P. (2023). Current Understanding of Antibiotic-Associated Dysbiosis and Approaches for Its Management. Ther. Adv. Infect. Dis..

[89] Sitkin S., Lazebnik L., Avalueva E., Kononova S., Vakhitov T. (2022). Gastrointestinal Microbiome and Helicobacter Pylori: Eradicate, Leave It as It Is, or Take a Personalized Benefit-Risk Approach?. World J. Gastroenterol..

[90] Chiu C.-Y., Chan Y.-L., Tsai M.-H., Wang C.-J., Chiang M.-H., Chiu C.-C. (2019). Gut Microbial Dysbiosis Is Associated with Allergen-Specific IgE Responses in Young Children with Airway Allergies. World Allergy Organ. J..

[91] Chiu C.-Y., Chan Y.-L., Tsai M.-H., Wang C.-J., Chiang M.-H., Chiu C.-C., Su S.-C. (2020). Cross-Talk between Airway and Gut Microbiome Links to IgE Responses to House Dust Mites in Childhood Airway Allergies. Sci. Rep..

[92] Inchingolo A.D., Inchingolo A.M., Malcangi G., Avantario P., Azzollini D., Buongiorno S., Viapiano F., Campanelli M., Ciocia A.M., De Leonardis N. (2022). Effects of Resveratrol, Curcumin and Quercetin Supplementation on Bone Metabolism—A Systematic Review. Nutrients.

[93] Bottalico L., Castellaneta F., Charitos I.A. (2020). From Hydrotherapy to The Discovery of The Gut Microbiota: The Historical Gastrointestinal Health Concept. Pharmacophore.

[94] Inchingolo A.D., Cazzolla A.P., Di Cosola P., Greco Lucchina A., Santacroce L., Charitos I.A., Topi S., Malcangi G., Hazballa D., Scarano A. (2021). The Integumentary System and Its Microbiota between Health and Disease. J. Biol. Regul. Homeost. Agents.

[95] Nagpal R., Mainali R., Ahmadi S., Wang S., Singh R., Kavanagh K., Kitzman D.W., Kushugulova A., Marotta F., Yadav H. (2018). Gut Microbiome and Aging: Physiological and Mechanistic Insights. Nutr. Healthy Aging.

[96] Newman H.N. (1974). Microbial Films in Nature. Microbios.

[97] Woodroffe R.C., Shaw D.A. (1974). Natural Control and Ecology of Microbial Populations on Skin and Hair. Soc. Appl. Bacteriol. Symp. Ser..

[98] Santacroce L., Charitos I.A., Ballini A., Inchingolo F., Luperto P., De Nitto E., Topi S. (2020). The Human Respiratory System and Its Microbiome at a Glimpse. Biology.

[99] Lovero R., Charitos I.A., Topi S., Castellaneta F., Cazzolla A.P., Colella M. (2024). Current Views About the Link between SARS-CoV-2 and the Liver: Friends or Foe?. Endocr. Metab. Immune Disord.-Drug Targets (Former. Curr. Drug Targets-Immune Endocr. Metab. Disord..

[100] Romano C., Cozzolino D., Nevola R., Abitabile M., Carusone C., Cinone F., Cuomo G., Nappo F., Sellitto A., Umano G.R. (2023). Liver Involvement During SARS-CoV-2 Infection Is Associated with a Worse Respiratory Outcome in COVID-19 Patients. Viruses.

[101] Milosevic I., Russo E., Vujovic A., Barac A., Stevanovic O., Gitto S., Amedei A. (2021). Microbiota and Viral Hepatitis: State of the Art of a Complex Matter. World J. Gastroenterol..

[102] Montagnani M., Bottalico L., Potenza M.A., Charitos I.A., Topi S., Colella M., Santacroce L. (2023). The Crosstalk between Gut Microbiota and Nervous System: A Bidirectional Interaction between Microorganisms and Metabolome. Int. J. Mol. Sci..

[103] Inchingolo A.D., Malcangi G., Semjonova A., Inchingolo A.M., Patano A., Coloccia G., Ceci S., Marinelli G., Di Pede C., Ciocia A.M. (2022). Oralbiotica/Oralbiotics: The Impact of Oral Microbiota on Dental Health and Demineralization: A Systematic Review of the Literature. Children.

[104] Kim N., Yun M., Oh Y.J., Choi H.-J. (2018). Mind-Altering with the Gut: Modulation of the Gut-Brain Axis with Probiotics. J. Microbiol..

[105] Liu X., Cao S., Zhang X. (2015). Modulation of Gut Microbiota-Brain Axis by Probiotics, Prebiotics, and Diet. J. Agric. Food Chem..

[106] Ashique S., Mohanto S., Ahmed M.G., Mishra N., Garg A., Chellappan D.K., Omara T., Iqbal S., Kahwa I. (2024). Gut-Brain Axis: A Cutting-Edge Approach to Target Neurological Disorders and Potential Synbiotic Application. Heliyon.

[107] You M., Chen N., Yang Y., Cheng L., He H., Cai Y., Liu Y., Liu H., Hong G. (2024). The Gut Microbiota-Brain Axis in Neurological Disorders. MedComm.

[108] Margolis K.G., Cryan J.F., Mayer E.A. (2021). The Microbiota-Gut-Brain Axis: From Motility to Mood. Gastroenterology.

[109] Bogusz K., Baran N., Maksymowicz M., Bielak A., Nowak A., Cywka Ł., Szwed W., Nowak A., Machowiec P. (2023). The Role of the Gut Microbiota in Pathogenesis and Treatment of Depression. J. Educ. Health Sport.

[110] Honarpisheh P., Bryan R.M., McCullough L.D. (2022). Aging Microbiota-Gut-Brain Axis in Stroke Risk and Outcome. Circ. Res..

[111] Carabotti M., Scirocco A., Maselli M.A., Severi C. (2015). The Gut-Brain Axis: Interactions between Enteric Microbiota, Central and Enteric Nervous Systems. Ann. Gastroenterol..

[112] Costa M., Brookes S.J., Hennig G.W. (2000). Anatomy and Physiology of the Enteric Nervous System. Gut.

[113] Goyal R.K. (2000). Targets of Enteric Motor Neurones: Smooth Muscle Cells. Gut.

[114] Rao M., Gershon M.D. (2018). Enteric Nervous System Development: What Could Possibly Go Wrong?. Nat. Rev. Neurosci..

[115] Lake J.I., Heuckeroth R.O. (2013). Enteric Nervous System Development: Migration, Differentiation, and Disease. Am. J. Physiol. Gastrointest. Liver Physiol..

[116] Inchingolo A.D., Malcangi G., Inchingolo A.M., Piras F., Settanni V., Garofoli G., Palmieri G., Ceci S., Patano A., De Leonardis N. (2022). Benefits and Implications of Resveratrol Supplementation on Microbiota Modulations: A Systematic Review of the Literature. Int. J. Mol. Sci..

[117] Browning K.N., Travagli R.A. (2014). Central Nervous System Control of Gastrointestinal Motility and Secretion and Modulation of Gastrointestinal Functions. Compr. Physiol..

[118] Altaf M.A., Sood M.R. (2008). The Nervous System and Gastrointestinal Function. Dev. Disabil. Res. Rev..

[119] Koenig J., Falvay D., Clamor A., Wagner J., Jarczok M.N., Ellis R.J., Weber C., Thayer J.F. (2016). Pneumogastric (Vagus) Nerve Activity Indexed by Heart Rate Variability in Chronic Pain Patients Compared to Healthy Controls: A Systematic Review and Meta-Analysis. Pain Physician.

[120] Lotufo P.A., Valiengo L., Benseñor I.M., Brunoni A.R. (2012). A Systematic Review and Meta-Analysis of Heart Rate Variability in Epilepsy and Antiepileptic Drugs. Epilepsia.

[121] Kenny B.J., Bordoni B. (2024). Neuroanatomy, Cranial Nerve 10 (Vagus Nerve). StatPearls.

[122] Baker E., Lui F. (2024). Neuroanatomy, Vagal Nerve Nuclei. StatPearls.

[123] Barbas-Henry H.A., Lohman A.H. (1984). The Motor Nuclei and Primary Projections of the IXth, Xth, XIth and XIIth Cranial Nerves in the Monitor Lizard, Varanus Exanthematicus. J. Comp. Neurol..

[124] Williams E.K., Chang R.B., Strochlic D.E., Umans B.D., Lowell B.B., Liberles S.D. (2016). Sensory Neurons That Detect Stretch and Nutrients in the Digestive System. Cell.

[125] Inchingolo A.D., Di Cosola M., Inchingolo A.M., Greco Lucchina A., Malcangi G., Pettini F., Scarano A., Bordea I.R., Hazballa D., Lorusso F. (2021). Correlation between Occlusal Trauma and Oral Microbiota: A Microbiological Investigation. J. Biol. Regul. Homeost. Agents.

[126] Stakenborg N., Gomez-Pinilla P.J., Verlinden T.J.M., Wolthuis A.M., D’Hoore A., Farré R., Herijgers P., Matteoli G., Boeckxstaens G.E. (2020). Comparison between the Cervical and Abdominal Vagus Nerves in Mice, Pigs, and Humans. Neurogastroenterol. Motil..

[127] Matteoli G., Boeckxstaens G.E. (2013). The Vagal Innervation of the Gut and Immune Homeostasis. Gut.

[128] Gargiulo Isacco C., Inchingolo A.D., Nguyen Cao K.D., Malcangi G., Paduanelli G., Pham Hung V., Tran Cong T., Bordea I.R., Scarano A., Laforgia A. (2021). The Bad Relationship, Osteo-Decay and Diabetes Type 2 Searching for a Link: A Literature Review. J. Biol. Regul. Homeost. Agents.

[129] Smith S.M., Vale W.W. (2006). The Role of the Hypothalamic-Pituitary-Adrenal Axis in Neuroendocrine Responses to Stress. Dialogues Clin. Neurosci..

[130] Chrousos G.P., Gold P.W. (1992). The Concepts of Stress and Stress System Disorders. Overview of Physical and Behavioral Homeostasis. JAMA.

[131] Jankord R., Herman J.P. (2008). Limbic Regulation of Hypothalamo-Pituitary-Adrenocortical Function during Acute and Chronic Stress. Ann. N. Y. Acad. Sci..

[132] Oyola M.G., Handa R.J. (2017). Hypothalamic-Pituitary-Adrenal and Hypothalamic-Pituitary-Gonadal Axes: Sex Differences in Regulation of Stress Responsivity. Stress.

[133] Abraham W.-R. (2016). Going beyond the Control of Quorum-Sensing to Combat Biofilm Infections. Antibiotics.

[134] Mittal R., Debs L.H., Patel A.P., Nguyen D., Patel K., O’Connor G., Grati M., Mittal J., Yan D., Eshraghi A.A. (2017). Neurotransmitters: The Critical Modulators Regulating Gut-Brain Axis. J. Cell. Physiol..

[135] Bamalan O.A., Moore M.J., Al Khalili Y. (2024). Physiology, Serotonin. StatPearls.

[136] Gao K., Mu C.-L., Farzi A., Zhu W.-Y. (2020). Tryptophan Metabolism: A Link Between the Gut Microbiota and Brain. Adv. Nutr..

[137] Zhou M., Fan Y., Xu L., Yu Z., Wang S., Xu H., Zhang J., Zhang L., Liu W., Wu L. (2023). Microbiome and Tryptophan Metabolomics Analysis in Adolescent Depression: Roles of the Gut Microbiota in the Regulation of Tryptophan-Derived Neurotransmitters and Behaviors in Human and Mice. Microbiome.

[138] Deng Y., Zhou M., Wang J., Yao J., Yu J., Liu W., Wu L., Wang J., Gao R. (2021). Involvement of the Microbiota-Gut-Brain Axis in Chronic Restraint Stress: Disturbances of the Kynurenine Metabolic Pathway in Both the Gut and Brain. Gut Microbes.

[139] Terry N., Margolis K.G. (2017). Serotonergic Mechanisms Regulating the GI Tract: Experimental Evidence and Therapeutic Relevance. Handb. Exp. Pharmacol..

[140] Aarsland T.I.M., Instanes J.T., Posserud M.-B.R., Ulvik A., Kessler U., Haavik J. (2022). Changes in Tryptophan-Kynurenine Metabolism in Patients with Depression Undergoing ECT—A Systematic Review. Pharmaceuticals.

[141] Juárez Olguín H., Calderón Guzmán D., Hernández García E., Barragán Mejía G. (2016). The Role of Dopamine and Its Dysfunction as a Consequence of Oxidative Stress. Oxid. Med. Cell. Longev..

[142] Wang P., Niu L., Gao L., Li W.X., Jia D., Wang X.L., Gao G.D. (2010). Neuroprotective Effect of Gypenosides against Oxidative Injury in the Substantia Nigra of a Mouse Model of Parkinson’s Disease. J. Int. Med. Res..

[143] (2010). Adrenaline and Noradrenaline.

[144] Byrne C.J., Khurana S., Kumar A., Tai T.C. (2018). Inflammatory Signaling in Hypertension: Regulation of Adrenal Catecholamine Biosynthesis. Front. Endocrinol..

[145] Paravati S., Rosani A., Warrington S.J. (2024). Physiology, Catecholamines. StatPearls.

[146] Cabana B.E., Prokesch J.C., Christiansen G.S. (1964). Study on the Biogenesis of Catecholamines in Pheochromocytoma Tissue Culture. Arch. Biochem. Biophys..

[147] Holzer P., Farzi A. (2014). Neuropeptides and the Microbiota-Gut-Brain Axis. Adv. Exp. Med. Biol..

[148] Dunn J., Grider M.H. (2024). Physiology, Adenosine Triphosphate. StatPearls.

[149] Nakrani M.N., Wineland R.H., Anjum F. (2024). Physiology, Glucose Metabolism. StatPearls.

[150] Lee J.H., Espinera A.R., Chen D., Choi K.-E., Caslin A.Y., Won S., Pecoraro V., Xu G.-Y., Wei L., Yu S.P. (2016). Neonatal Inflammatory Pain and Systemic Inflammatory Responses as Possible Environmental Factors in the Development of Autism Spectrum Disorder of Juvenile Rats. J. Neuroinflamm..

[151] Mezzelani A., Landini M., Facchiano F., Raggi M.E., Villa L., Molteni M., De Santis B., Brera C., Caroli A.M., Milanesi L. (2015). Environment, Dysbiosis, Immunity and Sex-Specific Susceptibility: A Translational Hypothesis for Regressive Autism Pathogenesis. Nutr. Neurosci..

[152] Silva Y.P., Bernardi A., Frozza R.L. (2020). The Role of Short-Chain Fatty Acids From Gut Microbiota in Gut-Brain Communication. Front. Endocrinol..

[153] Castro-Mejía J.L., Khakimov B., Aru V., Lind M.V., Garne E., Paulová P., Tavakkoli E., Hansen L.H., Smilde A.K., Holm L. (2022). Gut Microbiome and Its Cofactors Are Linked to Lipoprotein Distribution Profiles. Microorganisms.

[154] Fock E., Parnova R. (2023). Mechanisms of Blood–Brain Barrier Protection by Microbiota-Derived Short-Chain Fatty Acids. Cells.

[155] Kim G.-H., Shim J.-O. (2022). Gut Microbiota Affects Brain Development and Behavior. Clin. Exp. Pediatr..

[156] Di Cosola M., Cazzolla A.P., Charitos I.A., Ballini A., Inchingolo F., Santacroce L. (2021). Candida Albicans and Oral Carcinogenesis. A Brief Review. J. Fungi.

[157] Breit S., Kupferberg A., Rogler G., Hasler G. (2018). Vagus Nerve as Modulator of the Brain-Gut Axis in Psychiatric and Inflammatory Disorders. Front. Psychiatry.

[158] Clapp M., Aurora N., Herrera L., Bhatia M., Wilen E., Wakefield S. (2017). Gut Microbiota’s Effect on Mental Health: The Gut-Brain Axis. Clin. Pract..

[159] Kim Y.-K., Shin C. (2018). The Microbiota-Gut-Brain Axis in Neuropsychiatric Disorders: Pathophysiological Mechanisms and Novel Treatments. Curr. Neuropharmacol..

[160] Bastiaanssen T.F.S., Cowan C.S.M., Claesson M.J., Dinan T.G., Cryan J.F. (2019). Making Sense of … the Microbiome in Psychiatry. Int. J. Neuropsychopharmacol..

[161] Inchingolo A.D., Inchingolo A.M., Bordea I.R., Malcangi G., Xhajanka E., Scarano A., Lorusso F., Farronato M., Tartaglia G.M., Isacco C.G. (2021). SARS-CoV-2 Disease through Viral Genomic and Receptor Implications: An Overview of Diagnostic and Immunology Breakthroughs. Microorganisms.

[162] Binda S., Tremblay A., Iqbal U.H., Kassem O., Le Barz M., Thomas V., Bronner S., Perrot T., Ismail N., Parker J.A. (2024). Psychobiotics and the Microbiota–Gut–Brain Axis: Where Do We Go from Here?. Microorganisms.

[163] Santacroce L., Di Cosola M., Bottalico L., Topi S., Charitos I.A., Ballini A., Inchingolo F., Cazzolla A.P., Dipalma G. (2021). Focus on HPV Infection and the Molecular Mechanisms of Oral Carcinogenesis. Viruses.

[164] Kouli A., Torsney K.M., Kuan W.-L., Stoker T.B., Greenland J.C. (2018). Parkinson’s Disease: Etiology, Neuropathology, and Pathogenesis. Parkinson’s Disease: Pathogenesis and Clinical Aspects.

[165] Inchingolo A.D., Inchingolo A.M., Bordea I.R., Malcangi G., Xhajanka E., Scarano A., Lorusso F., Farronato M., Tartaglia G.M., Isacco C.G. (2021). SARS-CoV-2 Disease Adjuvant Therapies and Supplements Breakthrough for the Infection Prevention. Microorganisms.

[166] Roshan M.H.K., Tambo A., Pace N.P. (2016). Potential Role of Caffeine in the Treatment of Parkinson’s Disease. Open Neurol. J..

[167] Manfredsson F.P., Luk K.C., Benskey M.J., Gezer A., Garcia J., Kuhn N.C., Sandoval I.M., Patterson J.R., O’Mara A., Yonkers R. (2018). Induction of Alpha-Synuclein Pathology in the Enteric Nervous System of the Rat and Non-Human Primate Results in Gastrointestinal Dysmotility and Transient CNS Pathology. Neurobiol. Dis..

[168] Ma Q., Xing C., Long W., Wang H.Y., Liu Q., Wang R.F. (2019). Impact of Microbiota on Central Nervous System and Neurological Diseases: The Gut-Brain Axis. J. Neuroinflamm..

[169] Fung T.C. (2020). The Microbiota-Immune Axis as a Central Mediator of Gut-Brain Communication. Neurobiol. Dis..

[170] Spielman L.J., Gibson D.L., Klegeris A. (2018). Unhealthy Gut, Unhealthy Brain: The Role of the Intestinal Microbiota in Neurodegenerative Diseases. Neurochem. Int..

[171] Wang X., Liang Z., Wang S., Ma D., Zhu M., Feng J. (2022). Role of Gut Microbiota in Multiple Sclerosis and Potential Therapeutic Implications. Curr. Neuropharmacol..

[172] Chandra S., Alam M.T., Dey J., Sasidharan B.C., Ray U., Srivastava A.K., Gandhi S., Tripathi P.P. (2020). Healthy Gut, Healthy Brain: The Gut Microbiome in Neurodegenerative Disorders. Curr. Top. Med. Chem..

[173] Tankou S.K., Regev K., Healy B.C., Tjon E., Laghi L., Cox L.M., Kivisäkk P., Pierre I.V., Hrishikesh L., Gandhi R. (2018). A Probiotic Modulates the Microbiome and Immunity in Multiple Sclerosis. Ann. Neurol..

[174] Liu L., Wang H., Chen X., Zhang Y., Zhang H., Xie P. (2023). Gut Microbiota and Its Metabolites in Depression: From Pathogenesis to Treatment. EBioMedicine.

[175] Borovikova L.V., Ivanova S., Zhang M., Yang H., Botchkina G.I., Watkins L.R., Wang H., Abumrad N., Eaton J.W., Tracey K.J. (2000). Vagus Nerve Stimulation Attenuates the Systemic Inflammatory Response to Endotoxin. Nature.

[176] Johnston G.R., Webster N.R. (2009). Cytokines and the Immunomodulatory Function of the Vagus Nerve. Br. J. Anaesth..

[177] Yao M., Qu Y., Zheng Y., Guo H. (2024). The Effect of Exercise on Depression and Gut Microbiota: Possible Mechanisms. Brain Res. Bull..

[178] Santacroce L., Inchingolo F., Topi S., Del Prete R., Di Cosola M., Charitos I.A., Montagnani M. (2021). Potential Beneficial Role of Probiotics on the Outcome of COVID-19 Patients: An Evolving Perspective. Diabetes Metab. Syndr..

[179] Vafadari B. (2021). Stress and the Role of the Gut-Brain Axis in the Pathogenesis of Schizophrenia: A Literature Review. Int. J. Mol. Sci..

[180] Limbana T., Khan F., Eskander N. (2020). Gut Microbiome and Depression: How Microbes Affect the Way We Think—PubMed. Cureus.

[181] Santos J.M., Mathew M.S., Shah N., Pajuelo-Vasquez R., Mistry A.M., Heindl S.E. (2021). Pre and Post-Operative Alterations of the Gastrointestinal Microbiome Following Bariatric Surgery. Cureus.

[182] Mosquera F.E.C., Guevara-Montoya M.C., Serna-Ramirez V., Liscano Y. (2024). Neuroinflammation and Schizophrenia: New Therapeutic Strategies through Psychobiotics, Nanotechnology, and Artificial Intelligence (AI). J. Pers. Med..

[183] Qian X., Li Q., Zhu H., Chen Y., Lin G., Zhang H., Chen W., Wang G., Tian P. (2024). Bifidobacteria with Indole-3-Lactic Acid-Producing Capacity Exhibit Psychobiotic Potential via Reducing Neuroinflammation. Cell Rep. Med..

[184] Signorini L., Ballini A., Arrigoni R., De Leonardis F., Saini R., Cantore S., De Vito D., Coscia M.F., Dipalma G., Santacroce L. (2021). Evaluation of a Nutraceutical Product with Probiotics, Vitamin D, Plus Banaba Leaf Extracts (*Lagerstroemia speciosa*) in Glycemic Control. Endocr. Metab. Immune Disord. Drug Targets.

[185] Szeligowski T., Yun A.L., Lennox B.R., Burnet P.W.J. (2020). The Gut Microbiome and Schizophrenia: The Current State of the Field and Clinical Applications. Front. Psychiatry.

[186] Mallah K., Couch C., Borucki D.M., Toutonji A., Alshareef M., Tomlinson S. (2020). Anti-Inflammatory and Neuroprotective Agents in Clinical Trials for CNS Disease and Injury: Where Do We Go From Here?. Front. Immunol..

[187] Munawar N., Ahsan K., Muhammad K., Ahmad A., Anwar M.A., Shah I., Al Ameri A.K., Al Mughairbi F. (2021). Hidden Role of Gut Microbiome Dysbiosis in Schizophrenia: Antipsychotics or Psychobiotics as Therapeutics. Int. J. Mol. Sci..

[188] Maczurek A., Hager K., Kenklies M., Sharman M., Martins R., Engel J., Carlson D.A., Münch G. (2008). Lipoic Acid as an Anti-Inflammatory and Neuroprotective Treatment for Alzheimer’s Disease. Adv. Drug Deliv. Rev..

[189] Signorini L., De Leonardis F., Santacroce L., Haxhirexha K., Topi S., Fumarola L., Dipalma G., Coscia M.F., Inchingolo F. (2020). Probiotics May Modulate the Impact of Aging on Adults. J. Biol. Regul. Homeost. Agents.

[190] Gîlcă-Blanariu G.-E., Șchiopu C.G., Ștefănescu G., Mihai C., Diaconescu S., Afrăsânie V.A., Lupu V.V., Lupu A., Boloș A., Ștefănescu C. (2023). The Intertwining Roads between Psychological Distress and Gut Microbiota in Inflammatory Bowel Disease. Microorganisms.

[191] Flowers S.A., Ward K.M., Clark C.T. (2020). The Gut Microbiome in Bipolar Disorder and Pharmacotherapy Management. Neuropsychobiology.

[192] McGovern A.S., Hamlin A.S., Winter G. (2019). A Review of the Antimicrobial Side of Antidepressants and Its Putative Implications on the Gut Microbiome. Aust. N. Z. J. Psychiatry.

[193] Isacco C.G., Ballini A., De Vito D., Nguyen K.C.D., Cantore S., Bottalico L., Quagliuolo L., Boccellino M., Di Domenico M., Santacroce L. (2021). Rebalancing the Oral Microbiota as an Efficient Tool in Endocrine, Metabolic and Immune Disorders. Endocr. Metab. Immune Disord. Drug Targets.

[194] Shanahan F., Sheehan D. (2016). Microbial Contributions to Chronic Inflammation and Metabolic Disease. Curr. Opin. Clin. Nutr. Metab. Care.

[195] Bundgaard-Nielsen C., Knudsen J., Leutscher P.D., Lauritsen M.B., Nyegaard M., Hagstrøm S., Sørensen S. (2020). Gut Microbiota Profiles of Autism Spectrum Disorder and Attention Deficit/Hyperactivity Disorder: A Systematic Literature Review. Gut Microbes.

[196] Kurokawa S., Nomura K., Sanada K., Miyaho K., Ishii C., Fukuda S., Iwamoto C., Naraoka M., Yoneda S., Imafuku M. (2024). A Comparative Study on Dietary Diversity and Gut Microbial Diversity in Children with Autism Spectrum Disorder, Attention-Deficit Hyperactivity Disorder, Their Neurotypical Siblings, and Non-Related Neurotypical Volunteers: A Cross-Sectional Study. J. Child. Psychol. Psychiatry.

[197] Ballini A., Dipalma G., Isacco C.G., Boccellino M., Di Domenico M., Santacroce L., Nguyễn K.C.D., Scacco S., Calvani M., Boddi A. (2020). Oral Microbiota and Immune System Crosstalk: A Translational Research. Biology.

[198] Curran E.A., O’Neill S.M., Cryan J.F., Kenny L.C., Dinan T.G., Khashan A.S., Kearney P.M. (2015). Research Review: Birth by Caesarean Section and Development of Autism Spectrum Disorder and Attention-Deficit/Hyperactivity Disorder: A Systematic Review and Meta-Analysis. J. Child Psychol. Psychiatry.

[199] Curran E.A., Khashan A.S., Dalman C., Kenny L.C., Cryan J.F., Dinan T.G., Kearney P.M. (2016). Obstetric Mode of Delivery and Attention-Deficit/Hyperactivity Disorder: A Sibling-Matched Study. Int. J. Epidemiol..

[200] Xu M., Yu X., Fan B., Li G., Ji X. (2023). Influence of Mode of Delivery on Children’s Attention Deficit Hyperactivity Disorder and Childhood Intelligence. Psychiatry Investig..

[201] Matthews M., Nigg J.T., Fair D.A. (2014). Attention Deficit Hyperactivity Disorder. Curr. Top. Behav. Neurosci..

[202] Lange K.W., Reichl S., Lange K.M., Tucha L., Tucha O. (2010). The History of Attention Deficit Hyperactivity Disorder. Atten. Deficit Hyperact. Disord..

[203] Santacroce L., Sardaro N., Topi S., Pettini F., Bottalico L., Cantore S., Cascella G., Del Prete R., Dipalma G., Inchingolo F. (2020). The Pivotal Role of Oral Microbiota in Health and Disease. J. Biol. Regul. Homeost. Agents.

[204] Rogliani P., Calzetta L., Coppola A., Cavalli F., Ora J., Puxeddu E., Matera M.G., Cazzola M. (2017). Optimizing Drug Delivery in COPD: The Role of Inhaler Devices. Respir. Med..

[205] Ahmad S.I., Owens E.B., Hinshaw S.P. (2019). Little Evidence for Late-Onset ADHD in a Longitudinal Sample of Women. J. Consult. Clin. Psychol..

[206] Agnew-Blais J.C., Polanczyk G.V., Danese A., Wertz J., Moffitt T.E., Arseneault L. (2016). Evaluation of the Persistence, Remission, and Emergence of Attention-Deficit/Hyperactivity Disorder in Young Adulthood. JAMA Psychiatry.

[207] Kuddo T., Nelson K.B. (2003). How Common Are Gastrointestinal Disorders in Children with Autism?. Curr. Opin. Pediatr..

[208] Pulikkan J., Mazumder A., Grace T. (2019). Role of the Gut Microbiome in Autism Spectrum Disorders. Adv. Exp. Med. Biol..

[209] Luna R.A., Savidge T.C., Williams K.C. (2016). The Brain-Gut-Microbiome Axis: What Role Does It Play in Autism Spectrum Disorder?. Curr. Dev. Disord. Rep..

[210] Oh D., Cheon K.-A. (2020). Alteration of Gut Microbiota in Autism Spectrum Disorder: An Overview. J. Korean Acad. Child Adolesc. Psychiatry.

[211] Gilbert J.A., Blaser M.J., Caporaso J.G., Jansson J.K., Lynch S.V., Knight R. (2018). Current Understanding of the Human Microbiome. Nat. Med..

[212] Chuong K.H., Mack D.R., Stintzi A., O’Doherty K.C. (2018). Human Microbiome and Learning Healthcare Systems: Integrating Research and Precision Medicine for Inflammatory Bowel Disease. Omics J. Integr. Biol..

[213] Wang Q., Yang Q., Liu X. (2023). The Microbiota-Gut-Brain Axis and Neurodevelopmental Disorders. Protein Cell.

[214] Sha C., Jin Z., Ku S.Y., Kogosov A.S., Yu S., Bergese S.D., Hsieh H. (2024). Necrotizing Enterocolitis and Neurodevelopmental Impairments: Microbiome, Gut, and Brain Entanglements. Biomolecules.

[215] Lu J., Martin C.R., Claud E.C. (2023). Neurodevelopmental Outcome of Infants Who Develop Necrotizing Enterocolitis: The Gut-Brain Axis. Semin. Perinatol..

[216] Kaminski V.d.L., Michita R.T., Ellwanger J.H., Veit T.D., Schuch J.B., Riesgo R.D.S., Roman T., Chies J.A.B. (2023). Exploring Potential Impacts of Pregnancy-Related Maternal Immune Activation and Extracellular Vesicles on Immune Alterations Observed in Autism Spectrum Disorder. Heliyon.

[217] Njotto L.L., Simin J., Fornes R., Odsbu I., Mussche I., Callens S., Engstrand L., Bruyndonckx R., Brusselaers N. (2023). Maternal and Early-Life Exposure to Antibiotics and the Risk of Autism and Attention-Deficit Hyperactivity Disorder in Childhood: A Swedish Population-Based Cohort Study. Drug Saf..

[218] Avó-Baião R., Vareda R., Lopes A. (2024). Comment on: “Maternal and Early-Life Exposure to Antibiotics and the Risk of Autism and Attention-Deficit Hyperactivity Disorder in Childhood: A Swedish Population-Based Cohort Study”. Drug Saf..

[219] Oberlander T.F., Zwaigenbaum L. (2017). Disentangling Maternal Depression and Antidepressant Use During Pregnancy as Risks for Autism in Children. JAMA.

[220] Tran S.M.-S., Mohajeri M.H. (2021). The Role of Gut Bacterial Metabolites in Brain Development, Aging and Disease. Nutrients.

[221] Inchingolo F., Santacroce L., Cantore S., Ballini A., Del Prete R., Topi S., Saini R., Dipalma G., Arrigoni R. (2019). Probiotics and EpiCor® in Human Health. J. Biol. Regul. Homeost. Agents.

[222] Markowiak P., Śliżewska K. (2017). Effects of Probiotics, Prebiotics, and Synbiotics on Human Health. Nutrients.

[223] Johnson D., Letchumanan V., Thurairajasingam S., Lee L.-H. (2020). A Revolutionizing Approach to Autism Spectrum Disorder Using the Microbiome. Nutrients.

[224] Contaldo M., Lucchese A., Lajolo C., Rupe C., Di Stasio D., Romano A., Petruzzi M., Serpico R. (2021). The Oral Microbiota Changes in Orthodontic Patients and Effects on Oral Health: An Overview. J. Clin. Med..

[225] Arrigoni R., Ballini A., Santacroce L., Cantore S., Inchingolo A., Inchingolo F., Di Domenico M., Quagliuolo L., Boccellino M. (2022). Another Look at Dietary Polyphenols: Challenges in Cancer Prevention and Treatment. Curr. Med. Chem..

[226] Zhang J., Zhu G., Wan L., Liang Y., Liu X., Yan H., Zhang B., Yang G. (2023). Effect of Fecal Microbiota Transplantation in Children with Autism Spectrum Disorder: A Systematic Review. Front. Psychiatry.

[227] Ballini A., Paduanelli G., Inchingolo A., Nguyen K.C., Inchingolo A.M., van Pham H., Aityan S., Schiffman M., Tran T., Duy Huynh T. (2019). Bone Decay and beyond: How Can We Approach It Better. J. Biol. Regul. Homeost. Agents.

[228] Li Y., Xiao P., Cao R., Le J., Xu Q., Xiao F., Ye L., Wang X., Wang Y., Zhang T. (2024). Effects and Microbiota Changes Following Oral Lyophilized Fecal Microbiota Transplantation in Children with Autism Spectrum Disorder. Front. Pediatr..

